# Epidemiological Surveillance of Respiratory Diseases in Urban Stray Cats in Shanghai

**DOI:** 10.3390/ani14111562

**Published:** 2024-05-24

**Authors:** Dequan Yang, Houbin Ju, Xin Li, Haixiao Shen, Feifei Ge, Xianchao Yang, Hongjing Zhao, Xiujuan Wu, Xiaoying Zhu, Xiaoxu Wang, Jian Wang, Shixin Huang

**Affiliations:** Shanghai Animal Disease Control Center, Shanghai 201103, China; scadc_yang@163.com (D.Y.); chengzinono@126.com (H.J.); star880502@sina.com (X.L.); vet_shx@163.com (H.S.); qyfgff2369@aliyun.com (F.G.); yangxianchaoml@163.com (X.Y.); zhaohongjin945@163.com (H.Z.); 19512367689@163.com (X.W.); xyz_04072001@126.com (X.Z.); wangxiaoxu1129@163.com (X.W.)

**Keywords:** urban stray cats, respiratory tract pathogens, Feline herpes virus type I (FHV-1), Feline calicivirus (FCV), influenza A virus, *Bordetella bronchiseptica*, *Chlamydia felis*, *Mycoplasma felis*, epidemiological investigation

## Abstract

**Simple Summary:**

In order to understand the prevalence of respiratory diseases in urban stray cats in Shanghai and provide scientific evidence for the development of targeted prevention and control strategies for respiratory diseases in stray cats, we performed a molecular survey in Shanghai in 2022. A total of 374 stray cats were screened against six respiratory pathogens. Out of the 374 samples, 146 tested positive. *Mycoplasma felis* exhibited the highest positivity rate at 18.72% (70/374), followed by *Chlamydia felis* at 11.76% (44/374), feline calicivirus at 3.74% (14/374), feline herpesvirus type-1 at 3.48% (13/374), and *Bordetella bronchiseptica* at 1.34% (5/374); influenza A virus was not detected. *Mycoplasma felis* and *Chlamydia felis* were the primary causative agents of respiratory infections in urban stray cats, with *Mycoplasma felis* demonstrating a notably higher positivity rate compared to other respiratory pathogens and frequently co-infecting with *Chlamydia felis* and feline calicivirus. The positivity rate of *Mycoplasma felis* was elevated during the summer, autumn, and winter seasons, with no significant variance between seasons. These results highlight a substantial overall prevalence of respiratory pathogens in urban stray cats in the Shanghai region, characterized by seasonal patterns and concurrent infections with other pathogens. These findings suggest the need for comprehensive prevention and control measures to address respiratory pathogen infections in urban stray cats in the Shanghai area.

**Abstract:**

Urban stray cats are cats without owners that survive in the wild for extended periods of time. They are one of the most common stray animals in cities, and as such, monitoring the pathogens carried by urban stray cats is an important component of urban epidemiological surveillance. In order to understand the prevalence of respiratory diseases in urban stray cats in Shanghai and provide scientific evidence for the development of targeted prevention and control strategies for respiratory diseases in stray cats, we collected 374 ocular, nasal, and oropharyngeal swabs from urban stray cats in Shanghai from January 2022 to December 2022. After RNA extraction, we used real-time PCR to detect six respiratory pathogens, including influenza A virus, feline calicivirus, feline herpesvirus type 1, *Mycoplasma*, *Chlamydia*, and *Bordetella bronchiseptica*. The results showed that among the 374 samples, 146 tested positive, with a positivity rate of 39.04%. The highest positivity rate was observed for *Mycoplasma felis* at 18.72% (70/374), followed by *Chlamydia felis* at 11.76% (44/374), feline calicivirus at 3.74% (14/374), feline herpesvirus 1 at 3.48% (13/374), *Bordetella bronchiseptica* at 1.34% (5/374), and influenza A virus was not detected. The highest positivity rate for *Mycoplasma felis* was in Minhang District at 31.94% (23/72), while *Chlamydia felis* and *Bordetella bronchiseptica* had the highest positivity rates in Jiading District at 23.53% (8/34) and 5.88% (2/34), respectively. The highest positivity rates for feline calicivirus and feline herpesvirus 1 were both observed in Qingpu District, at 14.46% (12/83) and 9.64% (8/83), respectively. A total of 36 samples showed mixed infections with two or more pathogens, with *Mycoplasma felis* being involved in 32 of these mixed infections, with the highest number of mixed infections being with *Chlamydia felis* at 25 samples. Respiratory pathogen positivity was detected throughout the year, with peak detection rates in summer and winter. The positivity rates of cat respiratory pathogens in different seasons showed statistical differences (χ^2^ = 27.73, *p* < 0.01). There was no statistical difference in the positivity rates of respiratory pathogens between cats of different genders (χ^2^ = 0.92, *p* > 0.05). The positivity rates of respiratory pathogens in cats of different age groups showed statistical differences (χ^2^ = 44.41, *p* < 0.01). *Mycoplasma felis* and *Chlamydia felis* were the main pathogens causing respiratory infections in stray cats, with *Mycoplasma felis* showing a much higher positivity rate than other respiratory pathogens and often co-infecting with *Chlamydia felis* and feline calicivirus. The positivity rate of *Mycoplasma felis* was high in summer, autumn, and winter, with no statistical difference between seasons. These results indicate a serious overall prevalence of respiratory pathogens in urban stray cats in the Shanghai area, showing seasonal trends and mixed infections with other pathogens. These findings suggest the need for comprehensive prevention and control measures to address respiratory pathogen infections in urban stray cats in the Shanghai area.

## 1. Introduction

Upper respiratory tract disease (URTD) in cats is a common condition caused by one or more pathogens such as viruses and bacteria. Clinically, it manifests primarily as respiratory symptoms like coughing, sneezing, and nasal discharge, with severe cases leading to pneumonia and even death [1]. Common pathogens include Feline herpesvirus type-1 (FHV-1), Feline calicivirus (FCV), Influenza A virus (IV-A), *Bordetella bronchiseptica* (*B.b*), *Chlamydia felis* (*C. felis*), and *Mycoplasma felis* (*M. felis*) [2]. Clinical signs in URTD can be similar, regardless of the pathogen involved. FHV-1 typically causes upper respiratory and ocular disease. Corneal dendritic ulcers are considered pathognomonic for this infection [1]. FCV is an important pathogen that causes stomatitis, gingivitis, and tongue ulceration [3]. However, highly pathogenic FCVs have emerged, such as virulent systemic calicivirus (VS-FCV), which can cause a systemic viremic disease with fever, oral ulceration, skin ulceration, jaundice, and necrosis of the liver and other tissues, with a mortality rate as high as 67% [4]. In 2016, two H5N1 avian influenza virus strains were isolated from the lungs of stray cats exhibiting high fever, loss of appetite, and lethargy in Zhejiang Province, Eastern China [5]. So far, at least five subtypes of IV-A have been isolated from cats, including H1N1, H3N2, H5N1, H5N6, and H7N2 [2]. *B.b* can be isolated from apparently healthy cats and cats with respiratory disease, but it has been clearly associated with respiratory disease in cats [1]. *C. felis* mainly causes pneumonia and conjunctivitis in cats and is thought to be responsible for most cases of respiratory disease, together with FHV-1 and FCV [1]. *M. felis* is a normal resident of the upper respiratory tract and can be present as a commensal or a secondary pathogen, in association with conjunctivitis and URTD [6].

Stray animals, including domestic pets like cats and dogs, are animals that do not have owners [7]. They often live on their own, breed in an uncontrolled manner, and can cause problems by overpopulating their surroundings. This can pose challenges to public health and environmental safety on a global scale [8]. Specifically, urban stray cats are a notable animal population in urban areas, directly impacting environmental hygiene and human health [6,9,10,11]. Despite existing research indicating common pathogens such as FHV-1, FCV, *chlamydia*, and *mycoplasma* as primary causes of upper respiratory tract disease (URTD) in stray cats, there is a notable gap in understanding the epidemiology of URTD in urban stray cats in Shanghai. A comprehensive investigation into the epidemiological characteristics, transmission routes, and infection status of these pathogens is necessary to address this gap and inform effective interventions.

In this study, we performed an analysis of respiratory pathogens in 374 samples obtained from urban stray cats in Shanghai in 2022. The goal was to assess the prevalence of upper respiratory tract disease (URTD) in these cats, identify the primary pathogens causing URTD, and understand their distribution patterns. By doing so, we aimed to establish a scientific foundation for implementing specific measures to prevent and control feline diseases. Furthermore, our objective is to provide valuable scientific insights for epidemiological studies on respiratory illnesses in urban stray cats.

## 2. Materials and Methods

### 2.1. Samples Collection

In this study, samples were collected from January 2022 to December 2022, during the implementation of Trap–Neuter–Release (TNR) programs. Healthy urban stray cats without clinical respiratory symptoms were captured in eight districts. Basic information about these stray cats, including their origin, time of capture, age, sex, breed, etc., was collected. A total of 374 samples of ocular, nasal, and oropharyngeal swabs were collected and stored in sample preservation tubes containing 1 mL of 50% glycerol phosphate buffer, with samples from the three anatomical sites combined for nucleic acid extraction. All samples were procured in accordance with the Chinese legislation governing animal welfare and the guidelines stipulated by the Chinese policies and practices on veterinary medicine, with the explicit consent of the owners.

### 2.2. Reagents

The NucleoMag VET Bacteria Virus Nucleic Acid Extraction Kit was purchased from MN Company (Düren, Germany) in Germany. The Animal Influenza A Real-time RT-PCR Kit was obtained from Qiagen Bioengineering (Shenzhen) Co., Ltd. (Shenzhen, China) The One Step PrimeScript™ RT-PCR Kit (Perfect Real Time) and Premix Ex Taq™ (Probe qPCR) were purchased from Takara Biomedical Technology (Beijing) Co., Ltd. (Beijing, China).

### 2.3. Sample Treatment and Primers Designing

The tubes were vortexed in a shaker to release the unknown samples collected by the swabs into the solution. The liquid from the swabs was squeezed and the swabs were discarded. Then, the tubes were centrifuged at 3000 rpm at 4 °C for 5 min, and the supernatant was transferred to the corresponding 1.5 mL sterile centrifuge tubes. A 100 μL aliquot of the supernatant was used for nucleic acid extraction following the instructions of the NucleoMag VET Nucleic Acid Extraction Kit (Macherey-Nagel, Düren, Germany). The extracted nucleic acid was eluted in 100 μL of water and either immediately tested or stored at −70 °C for later use. Real-time TaqMan PCR primers and probes for FHV-1, FCV, *B.b*, *C. felis*, and *M. felis* were synthesized by referring to the previous studies [12,13] (Table 1). All primers and probes were synthesized by Shanghai Sunny Biotechnology Co., Ltd. (Shanghai, China).

### 2.4. Gene Amplification

A quantitative reverse transcriptase PCR (qRT-PCR) for IV-A was performed using the animal Influenza A Real-time RT-PCR Kit (Qiagen, Shenzhen, China) including positive and negative control, according to the manufacturer’s protocol. A qRT-PCR to detect FCV using One Step PrimeScript™ RT-PCR Kit (Perfect Real Time) (TakaRa, Beijing, China) was set up as follows: 12.5 µL of 2× One Step RT-PCR Buffer Ⅲ, 0.5 µL TaKaRa Ex Taq HS (5 U/μL), 0.5 μL PrimeScript RT Enzyme Mix Ⅱ, 0.5 μL each of FCV forward and reverse primers (10 μM), 1 µL FCV FAM-BHQ1 probe (10 μM), 5 µL FCV RNA and RNase-free water to 25 µL. The reaction was run in ABI Quant Studio 5 (Applied Biosystems, Foster City, CA, USA) and incubated at 42 °C for 5 min, 95 °C for 10 s and then for 45 cycles of 95 °C for five seconds and 60 °C for 30 s. Fluorescence was detected at 530 nm at each annealing step at 60 °C. Real-time PCR (qPCR) for FHV-1, *B.b*, *C. felis*, and *M. felis* were detected using Premix Ex Taq™ (Probe qPCR) (TakaRa, Beijing, China) as follows: Each reaction consisted of 12.5 µL of 2 × Premix Ex Taq (Probe qPCR), 0.5 μL each of forward and reverse primers (10 μM), 1 µL FAM-BHQ1 probe (10 μM), 5 µL DNA and RNase-free water to 25 µL. The reaction was run in ABI Quant Studio 5 (Applied Biosystems, Foster City, CA, USA) and incubated 95 °C for 30 s and then for 45 cycles of 95 °C for 5 s and 60 °C for 30 s. Fluorescence was detected at 530 nm at each annealing step at 60 °C. All qRT-PCR and qPCR data were analyzed using the ABI Quant Studio 5 software.

Positive and negative controls (RNase-free water) were included in the test samples. Positive controls consisted of viral strains including FCV (FCV/SH005/2018) and FHV-1 (FHV-1/SH010/2015); bacterial strains including *B.b* (*B.b*/SH-BS2/2020), *C. felis* (*C. felis*/SH-MH/2021) and *M. felis* (*M. felis*/SH-PT/2021). All these strains were isolated and identified at Shanghai Animal Disease Control Center (SCADC).

### 2.5. Data Analysis

Different software tools were used for data processing. Statistical analysis was performed using SPSS 22.0 software. Chi-square tests or Fisher’s exact probability tests were conducted for categorical data, with *p* < 0.05 considered statistically significant.

## 3. Results

### 3.1. Detection of Respiratory Pathogens among Urban Stray Cats

**Figure 1:**
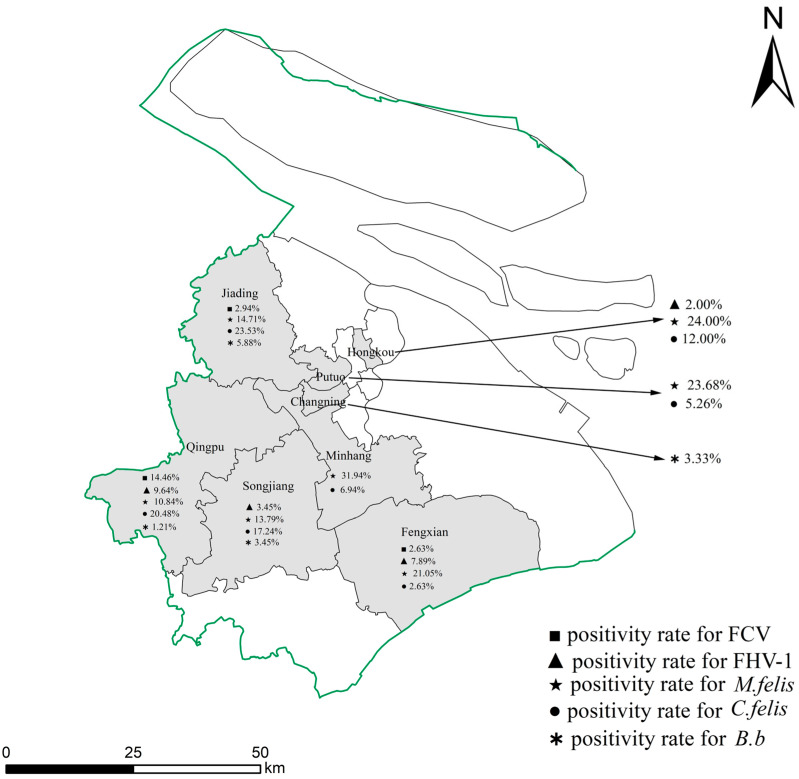
Geographical distribution of the eight districts with respiratory pathogens positivity rates in Shanghai. A total of 374 samples collected from eight districts in Shanghai were examined for the presence of IV-A, FCV, FHV-1, *M. felis*, *C. felis*, and *B.b*. Each symbol represents a pathogen, and the percentages provided indicate the proportion of positive samples for the specified pathogens in each district. The scale in the bottom-left corner represents the theoretical distances on the map.

Nucleic acid detection for IV-A, FCV, FHV-1, *M. felis, C. felis*, and *B.b* was conducted on all 374 samples from eight districts in Shanghai (Table 2). The results show that out of the 374 samples, 146 tested positive, resulting in a positivity rate of 39.04%. The highest positivity rate was observed for *M. felis* at 18.72% (70/374), followed by *C. felis* at 11.76% (44/374), FCV at 3.74% (14/374), FHV-1 at 3.48% (13/374), and *B.b* at 1.34% (5/374). IV-A was not detected. The highest positivity rate for *M. felis* was in Minhang District at 31.94% (23/72), while *C. felis* and *B.b* had the highest positivity rates in Jiading District at 23.53% (8/34) and 5.88% (2/34), respectively (Figure 1). The highest positivity rates for FCV and FHV-1 were both observed in Qingpu District at 14.46% (12/83) and 9.64% (8/83), respectively (Figure 1).

### 3.2. Co-Infections Status of Respiratory Pathogens among Urban Stray Cats

Out of the 374 samples, 36 cases of co-infections were identified, resulting in a co-infection rate of 9.63% (Table 3). Among these, 25 cases of co-infection with *M. felis* and *C. felis* were detected, with a positivity rate of 6.68% (25/374). Additionally, there were four cases of co-infection with FCV and *M. felis* (with a positivity rate of 1.07%), three cases with FCV and *C. felis* (with a positivity rate of 0.80%), one case with FHV-1 and *M. felis* (with a positivity rate of 0.27%), one case with *C. felis* and *B.b* (with a positivity rate of 0.27%), and two cases with FCV, *M. felis*, and *C. felis* (with a positivity rate of 0.53%). These results indicate the presence of mixed infections of upper respiratory tract pathogens in the population of urban stray cats.

### 3.3. The Seasonal Distribution Characteristics of Respiratory Pathogens among Urban Stray Cats

Different seasons’ respiratory pathogens were detected throughout the year (Table 4), with peaks in the summer and winter. The positivity rates were highest in the summer at 50.51% (50/99) and in the winter at 50.00% (59/118), followed by 25.00% (31/124) in autumn and 18.18% (6/33) in spring. We can see that there was a statistically significant difference in positivity rates among the seasons (χ^2^ = 27.73, *p* < 0.01). The peak detection of *M. felis* occurred in the summer, autumn, and winter, with the highest positivity rates in the summer at 22.22% (22/99), followed by 20.16% (25/124) in autumn, 16.95% (20/118) in winter, and 9.09% (3/33) in spring, with no statistically significant differences among the seasons (χ^2^ = 3.22, *p* > 0.05). *C. felis* showed peak detection in the summer and winter, with the highest positivity rates in winter at 18.64% (22/118) and in summer at 15.15% (15/99), followed by 6.06% (2/33) in spring and 4.03% (5/124) in autumn, with statistically significant differences among the seasons (χ^2^ = 15.34, *p* < 0.01). FCV had the highest positivity rate in the summer, while FHV-1 had the highest positivity rate in winter, with both pathogens undetected in spring.

### 3.4. The Distribution of Respiratory Pathogens among Male and Female Urban Stray Cats

As shown in Table 5, the positivity rates of respiratory pathogens in male and female urban stray cats were 41.57% (74 out of 178) and 36.73% (72 out of 196), respectively, indicating no statistically significant difference between the two genders (χ^2^ = 0.92, *p* > 0.05).

### 3.5. The Distribution of Respiratory Pathogens among Urban Stray Cats across Various Age Groups

When stratified by age, it was observed that urban stray cats aged 1 to 3 years had the highest respiratory pathogen positivity rate at 65.35% (Table 6). The positivity rates for respiratory pathogens in other age groups were as follows: ≤1 year old, 33.71% (60/178); ≥3 years old, 21.05% (20/95). There was a statistically significant difference among the age groups (χ^2^ = 44.41, *p* < 0.01). *M. felis*-positive urban stray cats were predominantly found in the 1- to 3-year-old age group, with positivity rates of 16.29% (29/178), 33.67% (34/101), and 7.37% (7/95) in the respective age groups. Statistical analysis revealed significant differences among the age groups (χ^2^ = 23.56, *p* < 0.01).

## 4. Discussion

The escalation of urbanization and increased population mobility have led to a gradual increase in the population of urban stray cats, presenting a potential threat to urban environmental and public health [14]. Shanghai, as a bustling international metropolis characterized by frequent human interactions, harbors a substantial urban stray cat population that directly influences urban environmental hygiene and human health. Despite this, there exists a research gap concerning the epidemiology of upper respiratory tract diseases (URTD) in urban stray cats within the Shanghai region. A thorough comprehension of the epidemiological characteristics, infection rates, and transmission pathways of pathogens can furnish a scientific foundation for formulating targeted prevention and control strategies [10,11].

FCV, FHV-1, *M. felis, C. felis*, and *B.b* are common pathogens causing respiratory infections in cats. This study focuses on the prevalent pathogens in the stray cat population, aiming to investigate their prevalence and seasonal distribution characteristics in the Shanghai region to support the development of targeted prevention and control measures. The study reveals that respiratory infections in urban stray cats occur throughout the year without distinct seasonal patterns, which is consistent with findings from studies on domestic cats [15,16]. The primary respiratory pathogens in infected urban stray cats are *M. felis* and *C. felis*, followed by FCV and FHV-1, differing from reports showing a higher FCV positivity rate compared to other pathogens in domestic cats [17]. Except for Changning District, where *M. felis* was not detected, the positivity rates for *M. felis* in other districts were all above 10%; similarly, except for Changning District, the positivity rates for *C. felis* in Jiading, Hongkou, Qingpu, and Songjiang districts were all above 10% (Figure 1). The high positivity rates of *M. felis* in all districts may be related to the living environment of stray cats. *Mycoplasma*, as an opportunistic pathogen, can invade the lower respiratory tract in stray cats with poor living conditions, leading to lower respiratory tract infections in the presence of other pathogenic viruses and bacteria, and often resulting in mixed infections with FHV-1, FCV, and *Chlamydia*. The study detected 70 cases of *M. felis* positivity, with 32 cases showing mixed infections with FCV, FHV-1, and *C. felis*, and its single positivity rate was significantly higher than that of other respiratory pathogens. In terms of the seasonal distribution of each pathogen, the positivity rate of *M. felis* was high in summer, autumn, and winter, with no statistical differences among seasons. There were statistically significant differences in *M. felis* positivity rates among urban stray cats aged ≤1 year, 1–3 years, and ≥3 years, and these differences were primarily distributed among younger cats.

In our study, the positivity rate of *C. felis* was higher in summer and winter, with statistical differences observed among seasons. According to a previous study [18], the prevalence of *C. felis* in healthy cats and cats without clinical symptoms is less than 2–3%. However, in this study, the positivity rate of *C. felis* was notably higher, reaching 11.76%. This could be attributed to the wide-ranging activities of urban stray cats and their frequent interactions, leading to the spread of infected cats carrying *C. felis*. Furthermore, once urban stray cats become infected with *C. felis*, it can result in persistent infections lasting up to 215 days. The results of this study indicate that mixed infections among urban stray cats with *M. felis, C. felis*, and FCV as the main pathogens are consistent with the conclusions drawn by Nguyen et al. [19], suggesting a relatively strong symbiotic relationship among these three pathogens.

In this study, FHV-1 showed a higher positivity rate in winter compared to summer, while FCV exhibited a higher positivity rate in summer than in winter, consistent with the findings reported by Gao et al. [20]. This variation may be related to the physical and chemical properties of the viruses themselves. FHV-1 is an enveloped DNA virus with poor heat resistance, while FCV is a non-enveloped RNA virus with strong resistance, being more tolerant to higher temperatures than FHV-1. Moreover, FCV is not sensitive to the antiviral effects of all disinfectants [21]. FCV infection generally occurs through direct contact with secretions from acutely infected and carrier cats. Moreover, indirect transmission can occur via fomites, which can contaminate in environment. FCV survives in the environment and remains infectious for up to one month on dry surfaces at room temperature, and even longer in colder conditions [3]. Disinfecting the environment with commercial sodium hypochlorite can reduce the spread of FCV [22]. The high positivity rate of *M. felis* in summer, autumn, and winter, consistent with the findings of Gao et al. [20], showed no statistical differences among seasons and primarily affected the 1–3-year-old cat population. According to the studies [23,24,25], *M. felis, C. felis*, and *B.b* pose potential threats to public health. Therefore, it is recommended to avoid contact with urban stray cats to prevent scratches, bites, and the risk of infection with *M. felis*, *C. felis*, and *B.b*. Additionally, enhanced monitoring of *M. felis*, *C. felis*, and *B.b* in urban stray cats is crucial for the prevention and control of respiratory diseases in stray cats, holding significant implications for public health safety. It is acknowledged that conducting a multivariable assessment of risk factors for disease severity is crucial during the epidemiological investigation of pathogens. Unfortunately, during our study, the COVID-19 pandemic in the Shanghai area had not completely subsided, which made it extremely challenging to collect samples that met the measured risk variables under the prevailing spatial and temporal conditions. Additionally, considering the potential transmission risk of SARS-CoV-2 among urban stray cat populations, we only sampled healthy urban stray cats without any clinical symptoms for our study and did not take into account factors related to risk variables. Despite the limitations in this work, our study largely updates the prevalence of upper respiratory tract pathogens in the healthy asymptomatic urban stray cat population in the Shanghai area.

## 5. Conclusions

This study aimed to investigate the occurrence of respiratory illnesses in urban stray cats in Shanghai and to furnish empirical support for the formulation of targeted measures for the prevention and management of respiratory diseases in this population. In 2022, we obtained 374 ocular, nasal, and oropharyngeal swabs from urban stray cats in Shanghai. Subsequent to RNA extraction, we utilized real-time PCR to identify six respiratory pathogens, namely Feline herpesvirus type-1 (FHV-1), Feline calicivirus (FCV), Influenza A virus (IV-A), *Bordetella bronchiseptica* (*B.b*), *Chlamydia felis* (*C. felis*), and *Mycoplasma felis* (*M. felis*). Out of the 374 samples, 146 tested positive. *Mycoplasma felis* exhibited the highest positivity rate at 18.72% (70/374), followed by *Chlamydia felis* at 11.76% (44/374), feline calicivirus at 3.74% (14/374), feline herpesvirus type-1 at 3.48% (13/374), and *Bordetella bronchiseptica* at 1.34% (5/374); influenza A virus was not detected. Additionally, we observed that *Mycoplasma felis* and *Chlamydia felis* were the primary causative agents of respiratory infections in urban stray cats, with *Mycoplasma felis* demonstrating a notably higher positivity rate compared to other respiratory pathogens and frequently co-infecting with *Chlamydia felis* and feline calicivirus. The positivity rate of *Mycoplasma felis* was elevated during the summer, autumn, and winter seasons, with no significant variance between seasons. These findings highlight a substantial overall prevalence of respiratory pathogens in urban stray cats in the Shanghai region, characterized by seasonal patterns and concurrent infections with other pathogens.

## Figures and Tables

**Table 1:** The PCR primers and probes involved in this study.

Primer	Sequence (5′ to 3′)	Size	References
FCV ORF1 gene	F: GTTGGATGAACTACCCGCCAATC R: CATATGCGGCTCTGATGGCTTGAAACTG P: FAM TCGGTGTTTGATTTGGCCTG BHQ1	121 bp	[12]
FHV-1 thymidine kinase gene	F: GGACAGCATAAAAGCGATTG R: AACGTGAACAACGACGCAG P: FAM AATTCCAGCCCGGAGCCTCAAT BHQ1	74 bp	[12]
*B.b* FimA	F: ACTATACGTCGGGAAATCTGTTTG R: CGTTGTCGGCTTTCGTCTG P: FAM CGGGCCGATAGTCAGGGCGTAG BHQ1	80 bp	[12]
*C. felis* major outer membrane protein gene	F: GAACTGCAAGCAACACCACTG R: CCATTCGGCATCTTGAAGATG P: FAM CGCTGCCGACAGATCAAATTTTGCC BHQ1	76 bp	[12]
*M. felis*	F: TAAATTAGCTCTTGATGGTGTTCCT R: TTCAAAGTCTTTTTCTGGAGTTTCA P: FAM TGAGAAGAAAAAGTTATGGAATTAATGGATGCA BHQ1	100 bp	[13]

Note: “F” is forward primer, “R” is reverse primer, “P” is probe.

**Table 2:** Detection results of IV-A, FCV, FHV-1, *M. felis*, *C. felis* and *B.b*.

District	Sample Numbers	Positive Number (Positive Rate)					
		IV-A	FCV	FHV-1	M. felis	C. felis	B.b
Fengxian	38	0 (0)	1 (2.63)	3 (7.89)	8 (21.05)	1 (2.63)	0 (0)
Minhang	72	0 (0)	0 (0)	0 (0)	23 (31.94)	5 (6.94)	0 (0)
Songjiang	29	0 (0)	0 (0)	1 (3.45)	4 (13.79)	5 (17.24)	1 (3.45)
Qingpu	83	0 (0)	12 (14.46)	8 (9.64)	9 (10.84)	17 (20.48)	1 (1.21)
Jiading	34	0 (0)	1 (2.94)	0 (0)	5 (14.71)	8 (23.53)	2 (5.88)
Changning	30	0 (0)	0 (0)	0 (0)	0 (0)	0 (0)	1 (3.33)
Hongkou	50	0 (0)	0 (0)	1 (2.00)	12 (24.00)	6 (12.00)	0 (0)
Putuo	38	0 (0)	0 (0)	0 (0)	9 (23.68)	2 (5.26)	0 (0)
Total	374	0 (0)	14 (3.74)	13 (3.48)	70 (18.72)	44 (11.76)	5 (1.34)

**Table 3:** The co-infection results of FCV, FHV-1, *M. felis*, *C. felis* and *B.b*.

Mixed Infection Pathogens	Sample Number	Positives Number	Positive Rate
FCV + *M. felis*	374	4	1.07
FCV + *C. felis*	374	3	0.80
FHV-1 + *M. felis*	374	1	0.27
*M. Felis* + *C. felis*	374	25	6.68
*C. Felis + B.b*	374	1	0.27
FCV + *M. felis* + *C. felis*	374	2	0.53
Total	374	36	9.63

**Table 4:** Seasonal distribution of urban stray cats infected with FCV, FHV-1, *M. felis*, *C. felis,* and *B.b*.

Pathogens	Seasons					χ^2^ Value	*p*-Value
	Spring (Mar.–May)	Summer (June–Aug.)	Autumn (Sept.–Nov.)	Winter (Dec.–Feb.)	Total		
	(*n* = 33)	(*n* = 99)	(*n* = 124)	(*n* = 118)	(*n* = 374)		
FCV	0 (0)	10 (10.10)	0 (0)	4 (3.39)	14 (3.74)	15.24 ^a^	<0.001
FHV-1	0 (0)	1 (1.01)	1 (0.81)	11 (9.32)	13 (3.48)	13.60 ^a^	0.001
*M. felis*	3 (9.09)	22 (22.22)	25 (20.16)	20 (16.95)	70 (18.72)	3.22	0.362
*C. felis*	2 (6.06)	15 (15.15)	5 (4.03)	22 (18.64)	44 (11.76)	15.34 ^a^	0.001
*B.b*	1 (3.03)	2 (2.02)	0 (0)	2 (1.69)	5 (1.34)	3.68 ^a^	0.251
Total	6 (18.18)	50 (50.51)	31 (25.00)	59 (50.00)	146 (39.04)	27.73	<0.001

Note: ^a^ is the Fisher’s exact test value.

**Table 5:** Gender distribution of urban stray cats infected with FCV, FHV-1, *M. felis*, *C. felis* and *B.b*.

Pathogens	Gender			χ^2^ Value	*p*-Value
	Male	Female	Total		
	(*n* = 178)	(*n* = 196)	(*n* = 374)		
FCV	6 (3.37)	8 (4.08)	14 (3.74)	0.13	0.790
FHV-1	5 (2.81)	8 (4.08)	13 (3.48)	0.45	0.580
*M. felis*	33 (18.54)	37 (18.88)	70 (18.72)	0.01	1.000
*C. felis*	26 (14.61)	18 (9.18)	44 (11.76)	2.64	0.111
*B.b*	4 (2.25)	1 (0.51)	5 (1.34)	1.02	0.313
Total	74 (41.57)	72 (36.73)	146 (39.04)	0.92	0.342

**Table 6:** Age distribution of urban stray cats infected with FCV, FHV-1, *M. felis*, *C. felis*, and *B.b*.

Pathogens	Age Groups				χ^2^ Value	*p*-Value
	≤1 Year Old	1~3 Years Old	≥3 Years Old	Total		
	(*n* = 178)	(*n* = 101)	(*n* = 95)	(*n* = 374)		
FCV	5 (2.81)	5 (4.95)	4 (4.21)	14 (3.74)	1.08 ^a^	0.645
FHV-1	4 (2.25)	6 (5.94)	3 (3.16)	13 (3.48)	2.56 ^a^	0.290
*M. felis*	29 (16.29)	34 (33.67)	7 (7.37)	70 (18.72)	23.56	<0.001
*C. felis*	20 (11.24)	19 (18.81)	5 (5.27)	44 (11.76)	8.75	0.012
*B.b*	2 (1.12)	2 (1.98)	1 (1.05)	5 (1.34)	0.68 ^a^	0.851
Total	60 (33.71)	66 (65.35)	20 (21.05)	146 (39.04)	44.41	< 0.001

Note: ^a^ is the Fisher’s exact test value.

## Data Availability

All data and results related to this study are included in the article. Raw data are available from the corresponding author upon reasonable request.
